# AGO Austria recommendations for genetic testing of patients with ovarian cancer

**DOI:** 10.1007/s00508-015-0814-7

**Published:** 2015-06-25

**Authors:** Christian Marth, Michael Hubalek, Edgar Petru, Stephan Polterauer, Alexander Reinthaller, Christian Schauer, Tonja Scholl-Firon, Christian F. Singer, Johannes Zschocke, Alain G. Zeimet

**Affiliations:** 1 Clinic for Gynecology and Obstetrics, Medical University of Innsbruck, Anichstrasse 35, 6020 Innsbruck, Austria; 2 Clinic for Gynecology and Obstetrics, Medical University of Graz, Graz, Austria; 3 Clinic for Gynecology, Clinical Department of General Gynecology and Gynecological Oncology, Medical University of Vienna, Vienna, Austria; 4 Department of Gynecology, Krankenhaus der Barmherzigen Brüder, Graz, Austria; 5 Department of Gynecology and Obstetrics, Wilhelminenspital, Vienna, Austria; 6 Division of Human Genetics, Medical University of Innsbruck, Innsbruck, Austria

**Keywords:** Ovarian cancer, *BRCA1*, *BRCA2*, Mutation, PARP, Ovarialkarzinom, *BRCA1*, *BRCA2*, Mutation, PARP

## Abstract

In Austria, 700 women are diagnosed every year with ovarian carcinoma. Approximately 15 % of the patients with epithelial ovarian cancer have a germline mutation in the *BRCA1* or *BRCA2* genes. The increased incidence of breast and/or ovarian cancer in genetically related family members has given rise to the term “hereditary breast and ovarian cancer syndrome” (HBOC). Some 25–55 % of these in-family diseases are attributed to germline mutations of *BRCA1* or *BRCA2*, and approximately 5–10 % to other known tumor predisposition syndromes. The remaining persons may carry mutations in as yet unidentified genes. HBOC caused by *BRCA1* and *BRCA2* mutations is an autosomal dominant disorder with high penetrance. *BRCA1* and *BRCA2* encode for so-called tumor suppressor proteins. Inherited functional mutations of these genes cause loss of function of the respective allele. Loss of function of the second allele causes complete loss of the corresponding protein and facilitates the development of a malignancy.

The Association of Gynecologic Oncology recommends that testing for a germline mutation in *BRCA1* or *BRCA2* should be offered to all patients with epithelial ovarian cancer. When mutations in *BRCA1, BRCA2,* or other cancer-susceptibility genes have been identified, patients with ovarian carcinoma can be treated with new, innovative therapies. This recommendation is intended as a standard guideline for genetic testing of patients with an ovarian carcinoma.

## Abstract (de)

In Österreich erkranken jedes Jahr etwa 700 Frauen an einem Ovarialkarzinom. Etwa 15 % der Patientinnen mit epithelialem Ovarialkarzinom sind Träger einer Keimbahnmutation im *BRCA1*- oder *BRCA2*-Gen. Aufgrund des häufig gemeinsamen Vorkommens von Mamma- und Ovarialkarzinomen spricht man vom „hereditären Mamma- und Ovarialkarzinomsyndrom“ (HBOC). Etwa 25–55 % dieser familiären Erkrankungen werden Keimbahnmutationen des *BRCA1*- oder *BRCA2*-Gens zugeschrieben, etwa 5–10 % anderen bekannten Tumordispositionssyndromen. Die verbleibenden Erkrankungen werden durch bisher nicht bekannte Gene erklärt. *BRCA1*- und *BRCA2*-Mutationen werden autosomal-dominant mit hoher Penetranz vererbt. Physiologischerweise kodieren *BRCA1* bzw. *BRCA2* für sogenannte Tumorsuppressorproteine. Funktionelle Mutationen dieser Gene führen zum Ausfall des Allels. Ein Ausfall auch des zweiten Allels führt zum Verlust der entsprechenden Proteine und erleichtert die maligne Transformation.

Eine genetische Testung und Bestimmung einer Keimbahnmutation in *BRCA1* oder *BRCA2* soll allen Patientinnen mit epithelialem Ovarialkarzinom angeboten werden. Durch den Nachweis von Mutationen im Bereich sogenannter Krebssuszeptibilitätsgene (wie *BRCA1* und *BRCA2*) können bei Patientinnen mit manifestem Ovarialkarzinom neue, innovative Therapien eingesetzt werden. Durch die vorliegende Empfehlung soll die genetische Testung von Patientinnen mit Ovarialkarzinom einheitlich definiert werden.

## Breast carcinomas and ovarian carcinomas

In its guideline issued in May 2011, the Austrian Society for Gynecology and Obstetrics took a standpoint on prevention and early diagnosis of breast and ovarian cancers in high-risk patients. From this recommendation, the indication for molecular genetic analysis of *BRCA1* and *BRCA2* was defined [1]:

**Indications for molecular genetic analysis of** *BRCA1* **and** *BRCA2*

**Table:** 

Two breast cancer cases before age of 50 years
Three breast cancer cases before age of 60 years
One breast cancer case before age of 35 years
One breast cancer case before age of 50 years and one ovarian cancer case at any age
Two ovarian cancer cases at any age
Male and female breast cancer at any age

This remains the basis for recommending genetic testing and meets the criteria for the cost to be paid by the Austrian national medical insurance system.

In Tyrol, Austria, the German S3 Guideline was incorporated into the recommendations of the Tyrolean Working Group for Clinical Oncology (TAKO) [2].

**TAKO recommendations:**

**Table:** 

**Only one affected person in a family**
Breast cancer with first occurrence before age of 35 years
Bilateral breast cancer with first occurrence before age of 50 years
Both breast and ovarian cancer at any age
**Multiple cases on the same side of a family**
Two women with breast cancer, one with first occurrence before age of 50 years
One woman with breast cancer and one woman with ovarian cancer
Two women with ovarian cancer
Three or more women with breast cancer
One man with breast cancer and one woman with breast or ovarian cancer

In general, a greater than 10 % prevalence of the mutation should be the threshold to recommend genetic testing. Even the very conservative National Institute for Health and Care Excellence recommends a genetic test at a calculated risk of 10 % or more [3].

Until now, the costs for genetic testing in patients with an ovarian carcinoma have been covered only when the family history was positive, which meant that additional family members usually must have been diagnosed with ovarian and/or breast cancer. Recent findings, however, have shown that up to 15 % of patients with epithelial ovarian cancer have germline mutations in *BRCA1* or *BRCA2* [4, 5]. Half of these patients have no apparent family history for ovarian cancer. This may be due, on the one hand, to the absence of female relatives, and on the other hand, to the insufficient knowledge of the family medical history.

Germline mutations in *BRCA1* or *BRCA2* are associated with early-onset breast cancer. For ovarian cancer, the correlation with age is less clear, as 35 % of the patients with hereditary ovarian cancer are of the age of 60 + years at the time of diagnosis.

In addition to germline mutations, at least another 2–8 % of ovarian cancers have somatic *BRCA1* and *BRCA2* mutations [5], so that up to 20 % of patients with ovarian cancer have a *BRCA1* or *BRCA2* deficiency triggered by a mutation. In addition, it is also known that 9–14 % of nonmutated ovarian cancers bear an epigenetic silencing of the *BRCA1* gene due to promoter hypermethylation [6].

Identification of a *BRCA* mutation has many consequences. One of them is that patients with a mutation-induced BRCA dysfunction have better survival and respond better to platinum therapy. Furthermore, a BRCA germline mutation is also associated with a risk for other tumors, particularly breast cancer. Such patients can decide to undergo an intensive early detection program or prophylactic surgery. Identification of a germline mutation also provides important information for other family members and their potential tumor risk, and permits personalized preventive measures to be outlined for high-risk persons. Genetic testing has taken on special importance through the introduction of new therapeutic possibilities using so-called “PARP (Poly ADP ribose polymerase) inhibitors” that are particularly effective in the case of a *BRCA1* or *BRCA2* mutation [7].

New technologies such as “next general sequencing” reduce the cost of genetic testing and permit additional genes to be tested. Blood samples taken in two GOG studies (GOG 218 and GOG 262) were subjected to genetic testing and showed a *BRCA1/2* mutation in 13.7 % of the patients with ovarian carcinoma, as well as mutations in other genes, in particular *BRIP1, PALB2, CHEK2, NBN,* and *ATM*, in 5.6 % of the patients [8]. The new testing methods pose special challenges for interpretation of the genetic data, knowledge of a wide range of cancer predispositions, and genetic counseling.

On the basis of these new findings, the Association of Gynecologic Oncology (AGO) Austria makes the following recommendations for women with ovarian cancer:

1. Genetic testing for *BRCA1* or *BRCA2* germline mutations should be offered to all patients with an epithelial ovarian carcinoma. Patients with borderline ovarian tumors or nonepithelial ovarian tumors and who do not meet the criteria for hereditary breast and ovarian carcinoma cannot be expected to benefit from testing.
2. Before germline mutation analysis of *BRCA1* and *BRCA2* in genomic DNA, the patient must undergo formal genetic counseling with regard to the possibility of a hereditary predisposition and must give her written consent for testing. The test results must be explained to the patient in a second personal genetic counselling session by a Medical Geneticist or a medical specialist for the particular indication as defined by the Austrian Gentechnikgesetz (GTG, Genetic Engineering Act). Counselling must be concluded with a counselling letter that contains all relevant points of the discussion, including the relevance of the findings for the patient’s family.
3. Before carrying out a genetic analysis of DNA isolated from tumor tissue that may potentially identify a germline mutation for example in *BRCA1* or *BRCA2*, the patient must be informed of the potential relevance of the test results for herself or other family members and must give her written consent for the test (for download see: AGO patient information sheet at: www.ago-austria.at).
4. If a probable germline mutation is identified in the tumor tissue, the test results must be conveyed to the patient by genetic counseling in compliance with the Austrian GTG. The patient must be informed about the relevance of the test results for herself and other family members as well as the possibilities for further work-up, and she must be offered the opportunity for germline mutation testing in genomic DNA (typically isolated from blood).
5. In view of the great significance for prognosis and treatment, especially, for possible PARP inhibitor therapy, not only a test for germline mutation, using DNA from a blood sample, but also quality-controlled testing of the tumor material should be performed. In an effort to personalize medicine, the AGO explicitly supports all innovative steps that promote the use of new technologies such as “next generation sequencing” to test for not only the two most common mutated genes, *BRCA1* and *BRCA2*, but also other genes involved in the homologous recombination repair of DNA as well as other relevant genes for ovarian cancer biology. In addition to testing solely for sequence alterations, assays should be developed in the future that also use, for example, epigenetic or functional analysis to better characterize tumor cells.

### Conflict of interest

The authors declare that there are no actual or potential conflicts of interest related to this article.
